# Elevation of effective p53 expression sensitizes wild-type p53 breast cancer cells to CDK7 inhibitor THZ1

**DOI:** 10.1186/s12964-022-00837-z

**Published:** 2022-09-05

**Authors:** Yueyuan Wang, Zhihao Zhang, Xuguang Mi, Mingxi Li, Dan Huang, Tingting Song, Xiaoyan Qi, Ming Yang

**Affiliations:** 1 Department of Breast Surgery, The First Hospital of Jilin University, Changchun, People’s Republic of China; 2 Tumor Biotherapy Center, Jilin Province People’s Hospital, Changchun, 130021 Jilin Republic of China; 3 Department of Neurology and Neuroscience Center, The First Hospital of Jilin University, Changchun, People’s Republic of China

**Keywords:** CDK7, p53, GSDME, THZ1, Breast cancer

## Abstract

**Background:**

The cyclin-dependent kinase 7 (CDK7) inhibitor THZ1 represses multiple cancer cells. However, its tumor-repressive efficiency in wild-type p53 breast cancer cells remains controversial.

**Methods:**

We conducted various assays, including CCK8, colony formation, flow cytometry, western blotting, and lactate dehydrogenase release detection, to clarify whether p53 elevation sensitizes breast cancer cells to THZ1.

**Results:**

We found that upregulating functional p53 contributes to the increased sensitivity of breast cancer cells to THZ1. Increased THZ1 sensitivity requires active p53 and an intact p53 pathway, which was confirmed by introducing exogenous wild-type p53 and the subsequent elevation of THZ1-mediated tumor suppression in breast cancer cells carrying mutant p53. We confirmed that p53 accumulates in the nucleus and mitochondria during cell death. Furthermore, we identified extensive transcriptional disruption, rather than solely CDK7 inhibition, as the mechanism underlying the nutlin-3 and THZ1-induced death of breast cancer cells. Finally, we observed the combined nutlin-3 and THZ1 treatment amplified gasdermin E cleavage.

**Conclusion:**

Enhanced sensitivity of breast cancer cells to THZ1 can be achieved by increasing effective p53 expression. Our approach may serve as a potential treatment for patients with breast cancer resistant to regular therapies.

**Video Abstract**

**Supplementary Information:**

The online version contains supplementary material available at 10.1186/s12964-022-00837-z.

## Introduction

Breast cancer is one of the most common tumors in women [1], and effective therapies are therefore of urgent necessity. Numerous treatments have been developed for breast cancer, including traditional surgical excision, chemotherapy, radiotherapy, immunotherapy, and targeted therapy. The introduction of therapeutic targeting represents significant progress in the decades-long battle against cancer. Most targeted drugs are directed against specific molecular targets that enable particular capabilities. Trastuzumab, for example, binds and blocks human epidermal growth factor receptor 2 expressed on the surface of cancer cells. Such affinity is considered an advantage, as it may be less toxic, and the specific inhibitory activity against a target implies fewer off-target effects. According to Hanahan and Weinberg, one of the hallmarks of cancer is evading growth suppressors, and cyclin-dependent kinase (CDK) inhibitors are designed to target this characteristic [2].

Eukaryotic cell division is controlled by CDKs that require CDK-activated kinase (CAK) to phosphorylate and activate [3]. CDK7, a CAK and an essential part of the general transcription factor IIH, regulates transcriptional processes by mediating the phosphorylation of RNA polymerase II and cell cycle division [4, 5]. THZ1 is a highly selective CDK7 inhibitor that binds to CDK7 outside the kinase domain at C132 residue. At high concentrations, it also inhibits the kinases CDK12 and CDK13 [6]. Moreover, it disrupts CDK7-dependent transcriptional addiction [7]. Because of its inhibitory effect on CDKs and modulatory roles in the transcription process, THZ1 potentially suppresses multiple cancers, such as bone marrow, pancreatic, liver, bile duct, lung, prostate, ovarian, and breast cancer [6, 8–12]. Although THZ1 has been demonstrated to suppress triple-negative breast cancer (TNBC), its anticancer effect on non-TNBC breast cancer cells, including MCF-7 and ZR-75-1 cells, is still controversial [12, 13]. We previously reported that a low THZ1 concentration mainly repressed MCF-7 cell proliferation rather than induced cell death [14, 15]. Therefore, strategies that decrease the survival and trigger the death of wild-type (WT) p53 breast cancer cells at low THZ1 concentrations might contribute to breast cancer therapy.

p53 is an extensively studied, well-known tumor-suppressor protein that functions as a transcription factor. It activates nearly 500 genes that control cell cycle arrest, cell senescence, DNA repair, metabolic adaptation, and cell death [16]. Thus, it mediates regulating the cell cycle, apoptosis, and several non-canonical cell death mechanisms. These include necroptosis, ferroptosis, autophagy, mitotic catastrophe, paraptosis, pyroptosis, and efferocytosis [17, 18]. Murine double minute 2 (MDM2), an E3 ligase, regulates p53 [19]. Nutlin-3 is a small-molecule MDM2 inhibitor that interferes with MDM2 and p53 binding. By occupying the p53-binding pocket of MDM2, nutlin-3 decreases p53 ubiquitination, promotes its accumulation, and activates the p53 signaling pathway [20–24].

Our previous study showed that the tumor-repressive effect of THZ1 was not significant in WT p53 breast cancer cells [14]. Here, we aimed to explore whether elevated p53 protein expression increases WT p53 breast cancer cell sensitivity to THZ1 or extensive transcriptional process abolishment, but not to CDK7 inhibition. A lethal effect was not observed in breast cancer cells with mutant-type (MT) p53 when treated with nutlin-3 and THZ1 combination, but the introduction of exogenous p53 rendered the cells more vulnerable to THZ1. Furthermore, different apoptosis-inducing pathways were initiated when p53 was located in different organelles. We conducted a series of assays to determine the site of p53 accumulation in WT p53 breast cancer cells treated with nutlin-3 and THZ1 and found the protein accumulating in the nucleus and mitochondria.

## Materials and methods

### Cell culture and treatment

Cells used in this study were purchased from the Cell Bank of the Type Culture Collection of the Chinese Academy of Sciences (Shanghai, China). 293 T, MCF-7, HS578T, HCC1937, and SK-BR-3 cells were cultured in DMEM (Hyclone, Logan, UT, USA) supplemented with 10% fetal bovine serum (Biological Industries, USA). MDA-MB-231 and DU4475 cells were cultured in RPMI medium (Hyclone, Logan, UT, USA) supplemented with 10% fetal bovine serum. ZR-75-1 cells were cultured in RPMI medium (Hyclone, Logan, UT, USA) supplemented with 20% fetal bovine serum. MCF10A cells were cultured in a breast epithelial cell complete medium provided by Zhong Qiao Xin Zhou Biotechnology Co, Ltd (Shanghai, China). All media contained 1% penicillin–streptomycin (Invitrogen, Carlsbad, CA, USA), and cells were cultured in a humidified 5% carbon dioxide atmosphere at 37 °C.

### Reagents and antibodies

THZ1 (HY-80013), flavopiridol (HY-10006), pifithrin β (HY-16702A), CX-5461 (HY-13323), and ML60218 ((HY-122122) were purchased from MedChemExpress (Monmouth Junction, NJ, USA). Nutlin-3 (S1061), LDC4297 (S7992), and triptolide (S3604) were obtained from Selleck (Houston, TX, USA). The primary antibodies used in the study were as follows: β-actin (Santa Cruz Biotechnology, Dallas, TX, USA); GSDME (Abcam, Cambridge, UK); CDK7, Tom 20, histone, PARP, and cleaved PARP (Cell Signaling Technology, Danvers, MA, USA); p53 (Proteintech, China). The secondary antibodies were anti-rabbit IgG (7074) and anti-mouse IgG (7076) (Cell Signaling Technology, Beverly, MA, USA). All the primary and secondary antibodies were diluted in TBS.

### Lentiviruses preparation

Plasmids pRSV-Rev (12,253), pMDLg/pRRE (12,251), and pCMV-VSV-G (8454) were purchased from Addgene (Cambridge, MA, USA). The lentiviral vector pLKO.1 was obtained from Generay Biotech (Shanghai, China). shRNA targeting CDK7 (sequence: 5-CCGGGCTGTAGAAGTGAGTTTGTAACTCGAGTTACAAACTCACTTCTACAGCTTTTT-3) was purchased from Sigma-Aldrich (St. Louis, MO, USA). The packaged viral vectors were added to the DMEM medium with 293 T cells. After 48 h, the supernatant of the 293 T cells was collected to harvest the recombinant lentivirus and purified through 0.45 µm membranes. Lentiviral transfection was performed to downregulate breast cancer cell-related proteins, as previously reported [14]. The effect of shRNA was assessed by western blotting.

### Transfection assay

MCF-7 cells were seeded in 6-well plates with 2 mL of medium. After reaching 70% confluence, they were transfected with a plasmid expressing the wild-type p53 gene using jetPRIME reagent (Polyplus-transfection, France). Approximately 5 μg/well of siRNA or 2 μg/well of plasmid was diluted with 200 μL/well buffer. The mixture was vortexed for 10 s, and the transfection reagent (4 μL/well) was added. The mixture was vortexed for 1 s and incubated for 10 min at room temperature. It was applied to the cells previously incubated for 48 h. Transfection was performed according to the manufacturer’s instructions and was assessed by western blotting.

### Cell viability assay

Cells were seeded at a density of 6,000 cells/well in 96-well plates containing 100 μL of the medium. After 24 h incubation to allow adherence, the medium was replaced with 100 μL of the fresh medium containing reagents at specific concentrations, and the cells were incubated further. For the cell viability assay of the transfection-treated cells, the relevant genes were down- or upregulated before seeding. Cell viability of the 6,000 cells/well, incubated in 100 μL of the appropriate medium, was assessed at 24, 48, and 72 h. Cells were incubated with 100 μL cell culture medium and 10 µL of CCK8 reagent (Abcam, Cambridge, UK) at 37 °C for 2 h before assessing OD. Absorbance was read at 450 nm using a BioTek ELISA reader (Winooski, VT, USA). The inhibition rates of the reagents on different cell lines were calculated according to the CCK8 assay results. The combination index (CI) of nutlin-3 + THZ1 in MCF-7, MDA-MB-231, and MCF10A cells was measured using CompuSyn software. CI values < 1 indicated synergy; CI = 1, addition; and CI > 1, antagonism [25]. All tests were independently repeated thrice.

### Colony formation assay

MCF-7, MDA-MB-231, and HS578T cells were seeded at approximately 6000 cells/well, and ZR-75-1, SK-BR-3, and HCC1937 cells were seeded at 200 000 cells/well with 2 mL medium in 6-well plates. The original medium was replaced after 24 h with 2 mL of the same medium containing different treatment concentrations or different drugs with appropriate concentrations, and the cells were then incubated for seven days. For the colony formation assay of MCF-7 cells subjected to CDK7 or p53 downregulation treatment, the relevant genes were downregulated prior to seeding, followed by the incubation of 6000 cells/well in 2 mL medium for seven days. For the colony formation assay of MCF-7 cells subjected to p53 upregulation treatment, relevant genes were upregulated prior to seeding and then 6000 cells/well were incubated in 2 mL medium for seven days. All cells used in the colony formation assay were washed with cold phosphate-buffered saline (PBS), fixed with 4% paraformaldehyde for 20 min, and stained with 0.1% crystal violet solution for 15 min at room temperature. The plates were then washed with double-distilled water and air-dried. The number of colonies on each plate was counted to assess cell colony formation. All tests were independently repeated three times.

### Apoptosis assay

MCF-7 cells were seeded at approximately 120,000 cells/well in 6-well plates containing 2 mL of the medium. After 24 h, it was replaced with 2 mL of the fresh medium containing reagents at different concentrations. After a further 24 h incubation, the cells were washed twice with PBS, gently trypsinized, and pelleted by centrifugation at 500 × *g* for 5 min at 4 °C. They were resuspended in cold PBS, collected by centrifugation at 500 × *g*, and resuspended in 100 μL cold binding buffer. Each group was treated with 5 μL annexin V-FITC and 5 μL PI and incubated with fluorochrome for 15 min at room temperature. Before flow cytometry, a binding buffer (400 μL) was added to each group to detect apoptosis. The Annexin V-FITC/PI Cell Apoptosis Detection Kit was purchased from TransGen (Beijing, China), and the flow cytometer was obtained from Thermo Fisher Scientific (USA).

For the apoptosis assay of MCF-7 cells subjected to CDK7 downregulation or transfection treatment, the relevant genes were downregulated or upregulated before seeding. The following procedures were the same as those used for the drug-treated group.

### Cell cycle analysis

MCF-7 or MDA-MB-231 cells were seeded in 6-well plates with a 2 mL medium. After 24 h, they were treated with 10 μM nutlin-3, 10 nM THZ1, or 10 μM nutlin-3 and 50 nM THZ1. The treated cells were harvested after 48 h, washed with cold PBS, and fixed with 70% cold ethanol overnight at 4 °C. The suspension was centrifuged at 1,000 × *g* for 7 min at 4 °C. The cell pellet was washed with cold PBS and collected following a further 7 min centrifugation at 1,000 × *g*. Each group was treated with 0.5 mL buffer, 10 μL of 50 × RNase, and 25 μL of 20 × PI. The cells were incubated with fluorochromes for 30 min at 37 °C before detection by flow cytometry.

### Isolation of nuclear proteins

MCF-7 cells were seeded at 12,000 cells/well in 6-well plates containing 2 mL of the medium. Cells were treated with drugs, washed twice with PBS, and lysed with low RIPA lysis buffer for 10 min on ice. The collected cell lysates were centrifuged at 14,000 × *g* for 15 min at 4 °C. The supernatant containing cytoplasmic proteins was transferred to a clean 1.5 mL microfuge tube and stored at − 80 °C to precipitate. A high RIPA buffer was added to the microfuge tube and left for 10 min to lyse the precipitate. The lysate was centrifuged at 14,000 × *g* for 15 min at 4 °C. The supernatant containing nuclear proteins was transferred to a new 1.5 mL microfuge tube and stored at − 80 °C.

### Isolation of mitochondria

MCF-7 cells were seeded at 720,000 cells/well in a 10 cm culture dish containing 12 mL medium and allowed to adhere for 24 h. The medium was replaced with 10 mL of the fresh medium containing the appropriate drug concentrations. Following a 48-h incubation, cells were washed twice with PBS, gently trypsinized, and pelleted by centrifugation at 1,000 rpm for 5 min at room temperature. The precipitates were resuspended in 400 μL of ice-cold MIB I buffer kept in 1.5 mL microfuge tubes. The tubes were incubated on ice for 2 min, and 5 μL/group of MIB II buffer was added. The mixtures were stirred for 5 s and incubated on ice for 5 min with vortexing every minute. The tubes were inverted five times after adding 400 μL/group of MIB III buffer. The supernatants were pelleted by centrifugation at 700 × *g* for 10 min at 4 °C and transferred into 2.0 mL microfuge tubes. After centrifugation at 12,000 × *g* for 15 min at 4 °C, the supernatants containing cytoplasmic proteins were stored at − 80 °C to precipitate. The precipitates were resuspended in 250 μL of MIB III buffer and centrifuged at 12,000 × *g* for 15 min at 4 °C. They were stored and lysed using RIPA buffer for blotting detection.

The procedure was performed according to the instructions of the ProteinExt Mammalian Mitochondria Isolation Kit for cultured cells (TransGen, China).

### Western blotting

MCF-7 cells were seeded at 120,000 cells/well in 6-well plates containing 2 mL medium and left to adhere for 24 h. The cells were treated with different reagent concentrations or transfected, washed twice with PBS, and lysed with RIPA lysis buffer for 10 min on ice. The collected cell lysates were centrifuged at 14,000 × *g* for 15 min at 4 °C. Protein levels were quantified using the Pierce BCA kit (Thermo Fisher Scientific, USA). Briefly, each sample included the supernatant and loading buffer preheated at 100 °C for 10 min. Samples were added at 20 μg/well, and proteins of different sizes were separated on 10% polyacrylamide gels. All proteins were transferred onto polyvinylidene fluoride membranes (Millipore, USA). The membranes were sliced into pieces, blocked with 5% skimmed milk for 1 h at room temperature, and incubated with primary antibody overnight at 4 °C. After 3 TBST washing cycles, the membranes were incubated with secondary antibodies at room temperature for 2 h. Protein expression levels were detected using the Pierce ECL reagent (Thermo Fisher Scientific, USA). All tests were independently repeated thrice.

### Lactate dehydrogenase release detection

MCF-7 cells were seeded at a density of 6,000 cells/well in 96-well plates containing 100 μL medium. After 24 h incubation to allow adherence, the medium was replaced with 100 μL of the fresh medium containing different reagent concentrations, and the cells were incubated further. LDH release was detected after 48 h using an LDH cytotoxicity assay kit (Beyotime, China). The LDH release reagent (10 μL/well) was added to the positive control group 1 h before the test. The medium containing the treated cells was collected in a 1.5 mL microfuge tube and centrifuged at 3,000 rpm for 5 min at 4 °C, and the supernatant was transferred to a clean 96-well plate. Each well was treated with 20 μL of lactic acid solution, 20 μL of enzyme solution, and 20 μL of 1 × INT solution. The mixture was incubated at room temperature for 30 min before measuring the OD. Absorbance was read at 492 nm using a BioTek ELISA reader (Winooski, VT, USA). All tests were independently repeated thrice.

### Statistical analysis

GraphPad Prism software (version 8.0; GraphPad Software, La Jolla, CA, USA) was utilized to visualize the data. Student’s t-test was used for statistical analyses to compare the differences between test groups. Statistical significance was set at *p* < 0.05. All experiments were performed in triplicates.

## Results

### Nutlin-3 elevates sensitivity of MCF-7 cells to THZ1

**Fig. 1:**
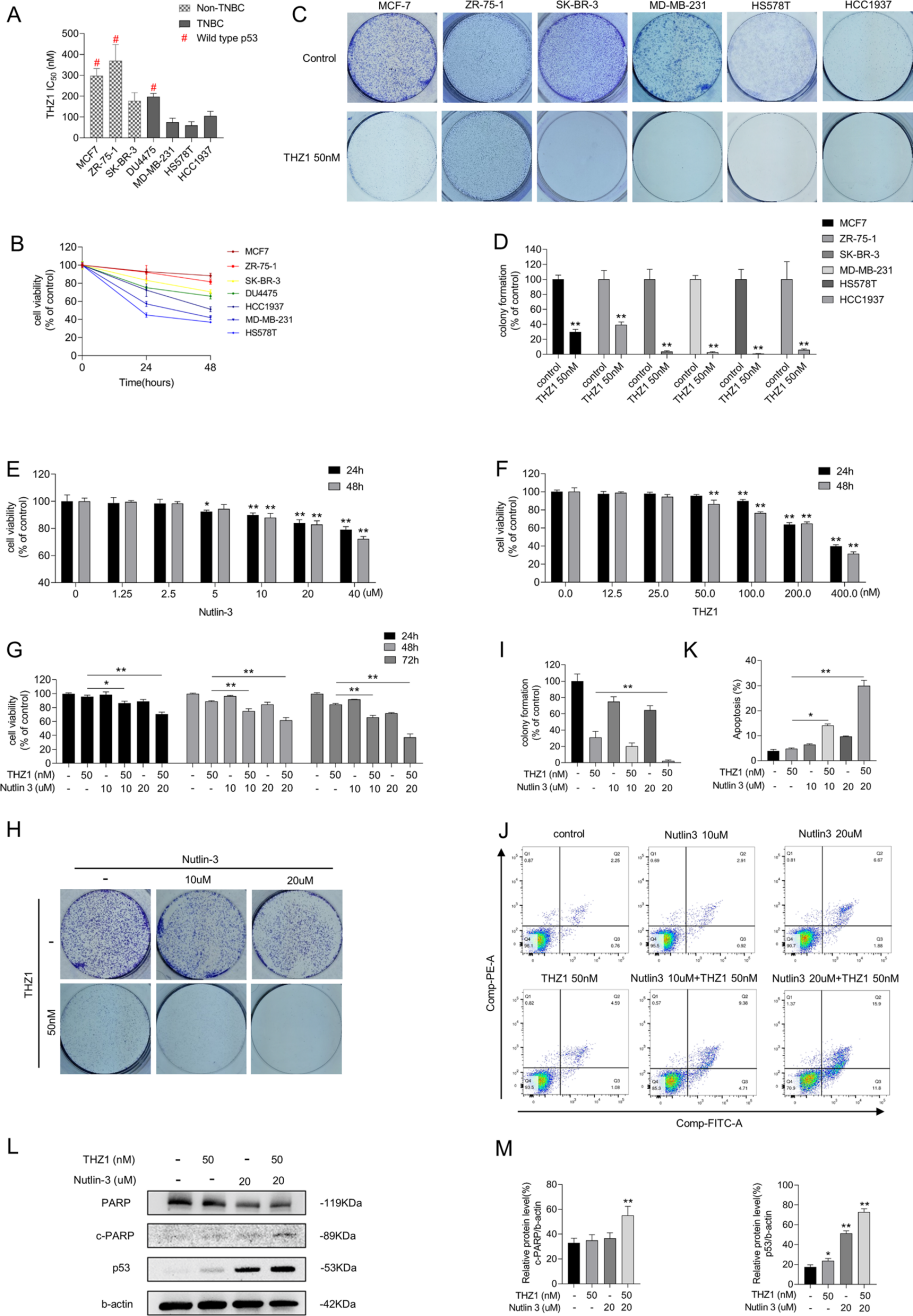
Nutlin-3 elevated the sensitivity of MCF-7 cells to THZ1. **A** Breast cancer cells were treated with increasing concentrations of THZ1 for 24 h and detected their cell viabilities by CCK8 assay. The IC50 values were calculated using GraphPad Prism software. **B** The cell viabilities of various breast cancer cells after treating with 50 nM THZ1 for 24 h and 48 h respectively were detected by CCK8 assay. **C** The colony formation ability was detected after breast cancer cells were treated with 50 nM THZ1. **D** Quantitation of colony formation ability in C. **E**, **F** The cell viability was detected by CCK8 assay after MCF-7 cell was treated with increasing concentrations of nutlin-3 or THZ1 for 24 h or 48 h separately. **G** The cell viability was detected by CCK8 assay after MCF-7 cell was treated with 10 nM or 20 nM nutlin-3 combining with 50 nM THZ1 for 24 h, 48 h or 72 h separately. **H** The colony formation ability was detected after MCF-7 cell was treated with 10 nM or 20 nM nutlin-3 combining with 50 nM THZ1. **I** Quantitation of colony formation ability in H. **J** Apoptosis was analyzed by flow cytometry after MCF-7 cell was treated with 10 nM or 20 nM nutlin-3 combining with 50 nM THZ1 for 48 h. **K** Quantity of apoptosis in J. **L** The protein levels of cleaved PARP and p53 were detected by western blot. **M** Quantity of cleaved PARP and P53 severally in L. Statistically significant (*P* < 0.05) are *, (*P* < 0.01) are **

Multiple breast cancer cell lines were used to verify the in vitro anti-tumorigenic effect of THZ1, a covalent molecular inhibitor of CDK7. As shown in Fig. 1A, TNBC cells had low IC50 values for THZ1 compared with hormone receptor-positive and HER2-positive breast cancer cells. A distinct lethal effect was detected in breast cancer cells with different p53 status. We separated the cell lines into two subtypes, TNBC cell lines (DU4475, MDA-MB-231, HCC1937, and HS578T) and non-TNBC cell lines (MCF-7, ZR-75-1, SK-BR-3), and treated them with 50 nM THZ1. Within the same subtype, cells carrying MT p53 were more sensitive to THZ1 than those expressing WT p53. Cells with MT p53, SK-BR-3, MDA-MB-231, HCC1937, and HS578T, were more sensitive to THZ1 than those without p53 mutations, MCF-7, ZR-75-1, and DU4475. Next, we determined cell viability and colony formation ability of the subtypes at different intervals on 50 nM THZ1. Consistent with the IC50 results, MCF-7 and ZR-750-1 cells expressing WT p53 were less sensitive to 50 nM THZ1 than SK-BR-3, MDA-MB-231, HS578T, and HCC1937 cells (Fig. 1B–D). Thus, although the suppressive effects of THZ1 on breast cancer cells are distinct, THZ1 is effective against a variety of breast cancer cell lines. The derivations, p53 status, type of p53 protein carried by the cells, their possible function, and other genomic alterations are listed in Table 1. Cell line derivations were obtained from the ATCC website (https://www.atcc.org), and P53 mutational status was obtained from the IARC TP53 Database (https://p53.iarc.fr/CellLines.aspx).

**Table 1:** Derivation, p53 and other genomic mutations of breast cancer cell lines used in this study

										p53 mutation					
Cell lines	Growth properties	Gene cluster	ER	PR	HER2	Tumor type	Age(years)	Ethnicity	P53 status	Nature	Site	cDNA discription	Protein	Functional impact	Other genomic alterations
MCF-7	Adherent	Luminal	+	+	–	Adenocarcinoma	69	white	WT						CDKN2A; PIK3CA
ZR-75–1	Adherent	Luminal	+	–	–	IDC	63	white	WT						–
SK-BR-3	Adherent	HER2 +	–	–	+	Adenocarcinoma	43	white	MUT	Missense mutations	5-exon	c.524G > A	p.R175H	Chemoresistance; Angiogenesis; Inflammatory response; Metabolic reprogramming; Genetic Instability; Tumor Cell proliferation; Migration, Invasion and Metastasis	–
DU4475	Suspension	TNBC	–	–	–	Carcinoma	70	white	WT						APC; BRAF; RB1
MB-231	Adherent	TNBC	–	–	–	Adenocarcinoma	51	white	MUT	Missense mutations	8-exon	c.839G > A	p.R280K	Chemoresistance; Angiogenesis; Inflammatory response	BRAF; CDKN2A; RAS
HS578T	Adherent	TNBC	–	–	–	Carcinoma	74	white	MUT	Missense mutations	5-exon	c.469G > T	p.V157F	A novel transcriptome regulation	–
HCC1937	Adherent	TNBC	–	–	–	IDC	23	white	MUT	Nonsense mutations	8-exon	c.916C > T	p.R306*		BRCA1

*ER* Estrogen receptor, *IDC* Invasive ductal carcinoma, *PR* Progestogen receptor, *TNBC* Triple-negative breast cancer, *WT* Wild type, *MUT* Mutant type

Kalan et al. reported that activating p53 transcription sensitizes colorectal cancer cells to CDK7 inhibitors [23]. We hypothesized that nutlin-3 would facilitate THZ1 to repress breast cancer cell survival. Initially, we treated MCF-7 cells with increasing concentrations of nutlin-3 or THZ1 but did not observe any significant tumor suppression (Fig. 1E, F). When MCF-7 cells were treated with gradient concentrations of nutlin-3, the effect on colony formation was insignificant at doses less than 40 µM (Additional file 1: Fig. S1A). Consistent with the synergistic lethality in HCT116 cells, lower cell viability, and fewer colony formation spots were observed only after treating MCF-7 cells with THZ1 and nutlin-3 combination (Fig. 1G–I). Flow cytometry results showed that the induction of cell death by 50 nM THZ1 in MCF-7 cells was low, whereas the apoptotic cell ratio increased after adding nutlin-3 to 50 nM THZ1 treatment (Fig. 1J, K). Furthermore, increased PARP cleavage and enhanced p53 expression were detected in the combined treatment (Fig. 1L, M). We also analyzed the effects of nutlin-3 and THZ1 on the MCF-7 cell cycle. The results showed that MCF-7 cells were arrested at the G1/S phase after nutlin-3 and THZ1 treatment (Additional file 1: Fig. S1B). To validate whether the combined treatment induces synthetic lethality, we treated MCF-7 and MCF-10A cells, a non-tumorigenic human mammary epithelial cell line, with different concentrations and proportions of nutlin-3 and THZ1 and calculated the CI (Additional file 1: Fig. S1C–F). The CI of nutlin-3 + THZ1 in MCF-7 cells was less than 1 and close to 1 in MCF-10A cells, implying that this combination synergistically suppressed MCF-7 cells. These results suggest that combined nutlin-3 and THZ1 induce significant anticancer effects and switch the cell fate from survival to death.

### Nutlin-3 elevates sensitivity of multiple breast cancer cell lines to THZ1

**Fig. 2:**
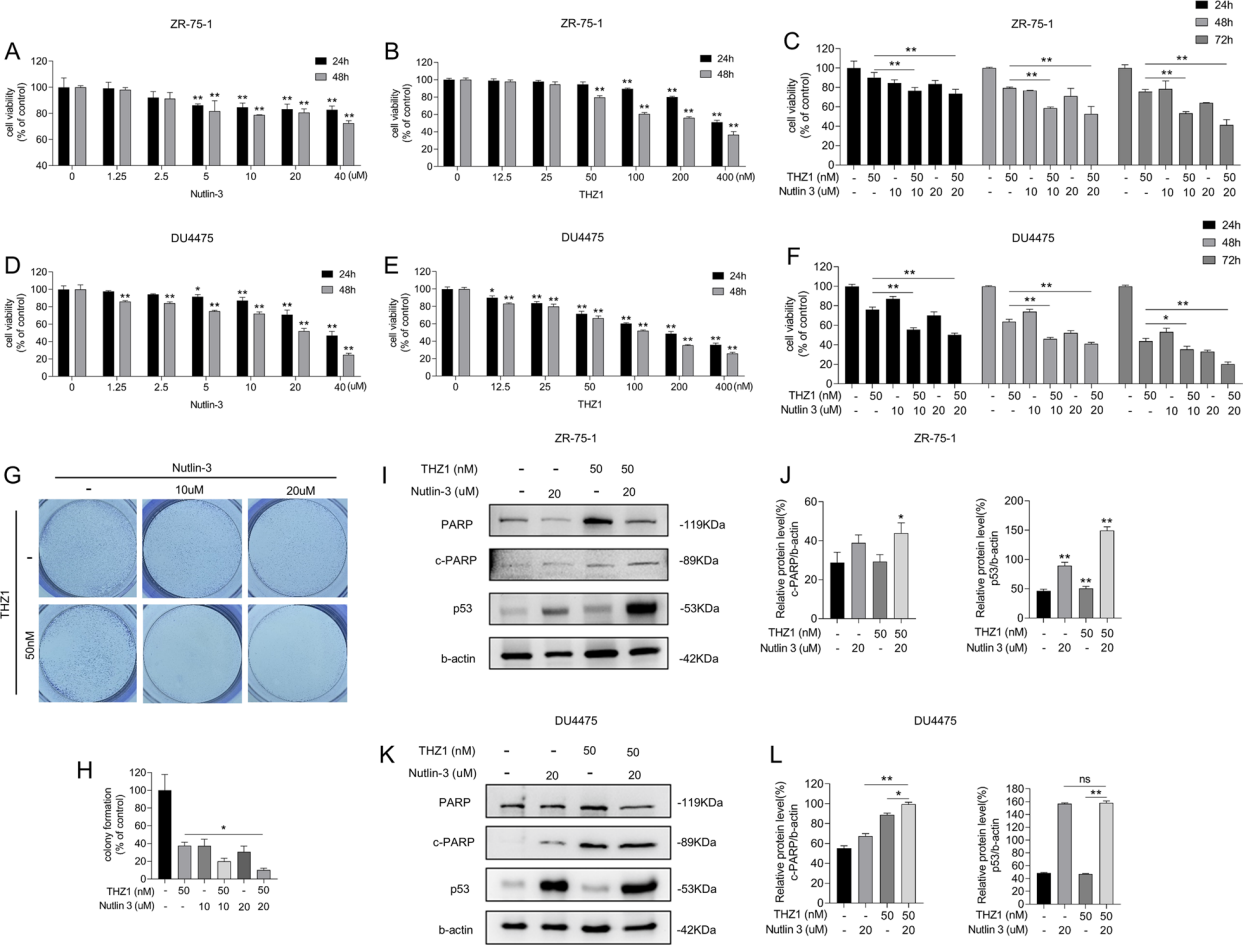
Nutlin-3 elevated the sensitivity of multiple breast cancer cell lines to THZ1. **A**, **D** CCK8 assay was utilized to measure the viability of ZR-75-1 or DU4475 cells with increasing concentrations of nutlin-3 for 24 h or 48 h. **B**, **E** CCK8 assay was utilized to measure the viability of ZR-75-1 or DU4475 cells with increasing concentrations of THZ1 for 24 h or 48 h. **C**, **F** The cell viability was detected by CCK8 assay after ZR-75-1 or DU4475 cells were treated with 10 nM or 20 nM nutlin-3 combining with 50 nM THZ1 for 24 h, 48 h or 72 h separately. **G** The colony formation assay was meant to test the ability of ZR-75-1 cell with 10 nM or 20 nM nutlin-3 combining with 50 nM THZ1 to form colony spots. **H** Quantitation of colony formation ability in G. **I** The protein levels of cleaved PARP and p53 in ZR-75-1 cell were detected by western blot. **J** Quantity of cleaved PARP and P53 severally in I. **K** The protein levels of cleaved PARP and p53 in DU4475 cell were detected by western blot. **L** Quantity of cleaved PARP and P53 severally in K. Statistically significant (*P* < 0.05) are *, (*P* < 0.01) are **

We conducted a CCK8 assay to evaluate the anticancer effect of nutlin-3 and THZ1 in ZR-75-1 and DU4475 cell lines. The results showed that treating ZR-75-1 and DU4475 with nutlin-3 or THZ1 alone produced a weaker anticancer effect than in combination (Fig. 2A–F). Moreover, the ZR-75-1 cell colony formation assay confirmed the THZ1 suppressive effect (Fig. 2G, H). In agreement with MCF-7 cells, p53 expression and PARP cleavage were elevated on nutlin-3 and THZ1 treatment in ZR-75-1 and DU4775 cells (Fig. 2I–L). Therefore, the antitumor effects of combined nutlin-3 and THZ1 are likely universal in WT p53 breast cancer cells.

### Increased p53 expression augments cytotoxicity of THZ1

**Fig. 3:**
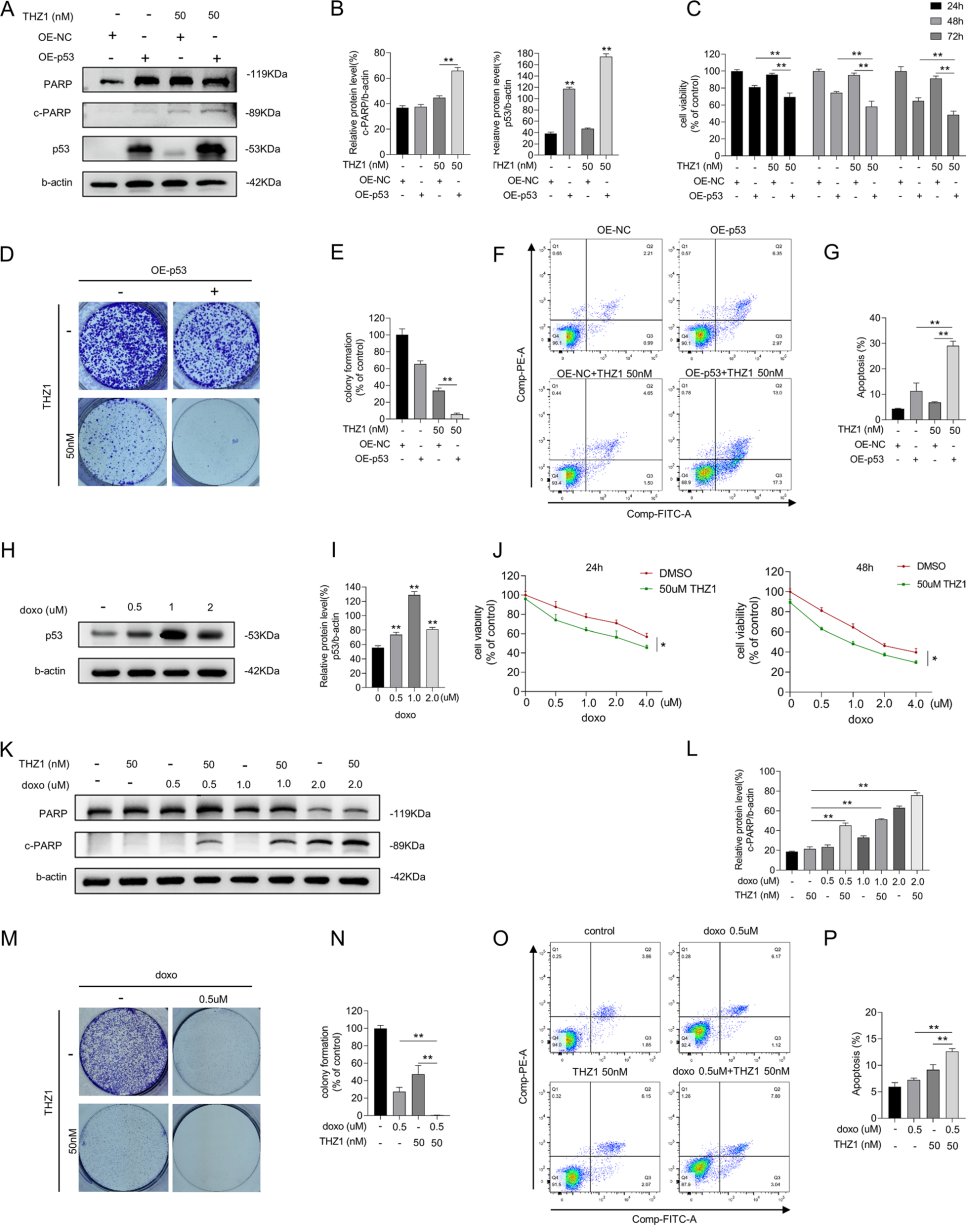
Increase p53 expression augmented cytotoxicity of THZ1. **A** The protein levels of cleaved PARP and p53 were detected by western blot. **B** Quantity of cleaved PARP and p53 in A. **C** Different time-point cell viability of MCF-7 cell after transfecting WT p53 plasmids and plasmids of control, then treated with 50 nM THZ1 was measured by CCK8 assay. **D** Colony formation ability measurement of MCF-7 cell after transfecting WT p53 plasmids or plasmids of control, then added with 50 nM THZ1. **E** Quantitation of colony formation ability in D. **F **Apoptosis was analyzed by flow cytometry after MCF-7 cells that transfected with WT p53 plasmids or plasmids of control were treated with 50 nM THZ1 for 48 h. **G** Quantity of apoptosis in F. **H** Western blot was utilized to verify p53 expression in MCF-7 cell under increasing concentrations of doxorubicin. **I** Quantity of p53 in H. **J** The cell viabilities of MCF-7 cell after treating with increasing concentrations of doxorubicin combining with 50 nM THZ1 or DMSO for 24 h and 48 h respectively were detected by CCK8 assay. **K** The protein level of cleaved PARP was detected by western blot. **L** Quantity of cleaved PARP in K. **M** Colony formation ability measurement of MCF-7 cell treated with 50 nM THZ1 and 0.5uM doxorubicin. **N** Quantitation of colony formation ability in M. **O** Apoptosis was analyzed by flow cytometry after MCF-7 cells treated with 50 nM THZ1 and 0.5uM doxorubicin for 24 h. **P** Quantity of apoptosis in O. Statistically significant (*P* < 0.05) are *, (*P* < 0.01) are **

As nutlin-3 elevates p53 by avoiding ubiquitination of MDM2, we verified whether increasing p53 expression without MDM2 intervention could enhance apoptotic cell rates. We overexpressed the WT p53 gene in MCF-7 cells and added THZ1 to p53 pre-upregulated cells. A blotting assay was performed to estimate the upregulation and induction of PARP cleavage (Fig. 3A, B). As shown in Figs. 3C–E, viability and colony formation ability of the cells were decreased. Overexpressing p53 also amplified the lethal effects of THZ1 on MCF-7 cells (Fig. 3F, G). Hence, MCF-7 cells overexpressing p53 display greater sensitivity to THZ1 than cells transfected with control plasmids.

Doxorubicin, a first-line chemotherapeutic drug for breast cancer therapy, increased p53 expression by damaging the DNA (Fig. 3H, I). We treated MCF-7 cells with doxorubicin and THZ1 and determined cell viability, colony formation, and apoptotic cell rate. We found that the combined treatment was more effective in reducing cancer cell survival than doxorubicin or THZ1 alone (Fig. 3J, M–P). Moreover, PARP cleavage was also enhanced (Fig. 3K, L). These data indicate that upregulating p53 facilitates the anticancer role of THZ1 in breast cancer cells.

### Nutlin-3 and THZ1-mediated lethality is p53-dependent

**Fig. 4:**
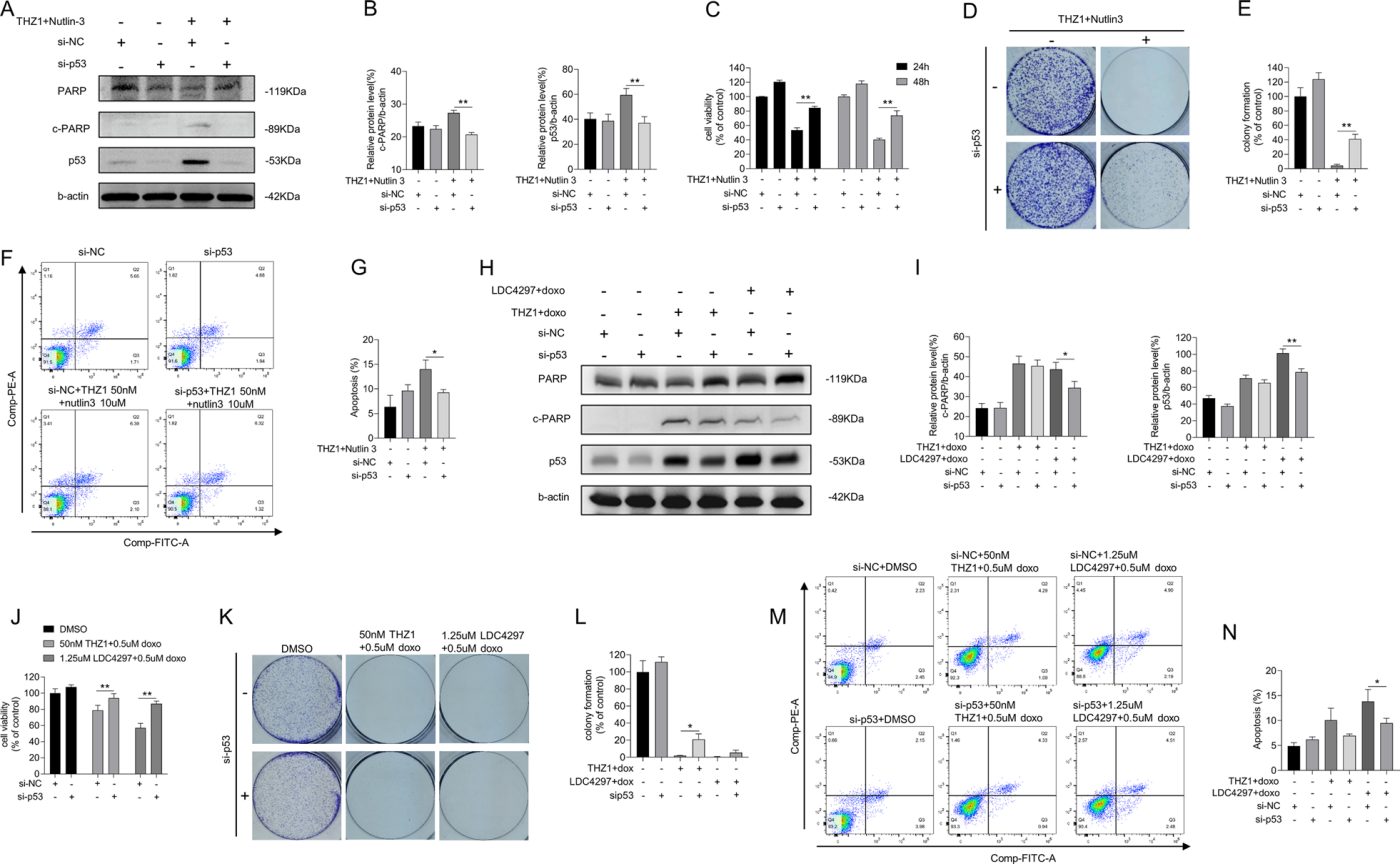
Lethality mediated by nutlin-3 + THZ1 required for p53 expression. **A** si-p53 was transfected into MCF-7 cells previously which would following be given 50 M THZ1and 10uM nutlin-3 treatment for 48 h. Western blot was conducted to display the expression of PARP, cleaved PARP, p53 and b-actin in MCF-7 cell. **B** Quantity of cleaved PARP and p53 in A. **C** CCK8 assay was used to measure the cell viability of MCF-7 cell treated with si-p53 or si-control combining 50 M THZ1and 10uM nutlin-3 for 24 h and 48 h. **D** Colony formation ability measurement of MCF-7 cell after transfecting si-p53 or si-control, then added with 50 nM THZ1 + 10uM nutlin-3. **E** Quantitation of colony formation ability in D. **F** Apoptosis was analyzed by flow cytometry after MCF-7 cells that transfected with si-p53 or si-control were treated with 50 nM THZ1 + 10uM nutlin-3 for 48 h. **G** Quantity of apoptosis in F. **H** Western blot was utilized to detect PARP, cleaved PARP, p53 and b-actin protein expression in MCF-7 cells which were treated with si-p53 combining 50 nM THZ1 + 0.5uM doxorubicin or 1.25uM LDC4297 + 0.5uM doxorubicin. **I** Quantity of cleaved PARP and p53 in H. **J** CCK8 assay was used to measure the cell viability of MCF-7 cells treated with si-p53 combining 50 nM THZ1 + 0.5uM doxorubicin or 1.25uM LDC4297 + 0.5uM doxorubicin for 24 h. **K** Colony formation ability measurement of MCF-7 cell after transfecting si-p53 or si-control, then added with 50 nM THZ1 + 0.5uM doxorubicin or 1.25uM LDC4297 + 0.5uM doxorubicin. **L** Quantitation of colony formation ability in K. **M** Apoptosis was analyzed by flow cytometry after MCF-7 cells that transfected with si-p53 or si-control were followed by 50 nM THZ1 + 0.5uM doxorubicin or 1.25uM LDC4297 + 0.5uM doxorubicin treatment for 24 h. **N** Quantity of apoptosis in M. Statistically significant (*P* < 0.05) are *, (*P* < 0.01) are **

The expression of p53 after combined nutlin-3 and THZ1 treatment was visibly higher than that in MCF-7 cells treated with THZ1 or nutlin-3 alone (Fig. 1L). We proceeded to verify whether p53 is required for this tumor-suppressive induction. We used siRNAs to interfere with p53 transcription and confirmed the decrease in p53 protein levels by immunoblotting (Fig. 4A, B). The cell group that received si-p53 combined with nutlin-3 and THZ1 treatment exhibited rapid proliferation, better colony formation ability, and lower apoptotic ratio than the si-control group treated only with nutlin-3 and THZ1 (Fig. 4C–G). These results were verified by combining doxorubicin with THZ1 or LDC4297. LDC4297 is also a CDK7 inhibitor that functions at nanomolar concentrations. Consistent with nutlin-3 and THZ1 treatment, the anticancer effects of CDK7 inhibitors combined with doxorubicin decreased when p53 expression was compromised (Fig. 4H–N).

**Fig. 5:**
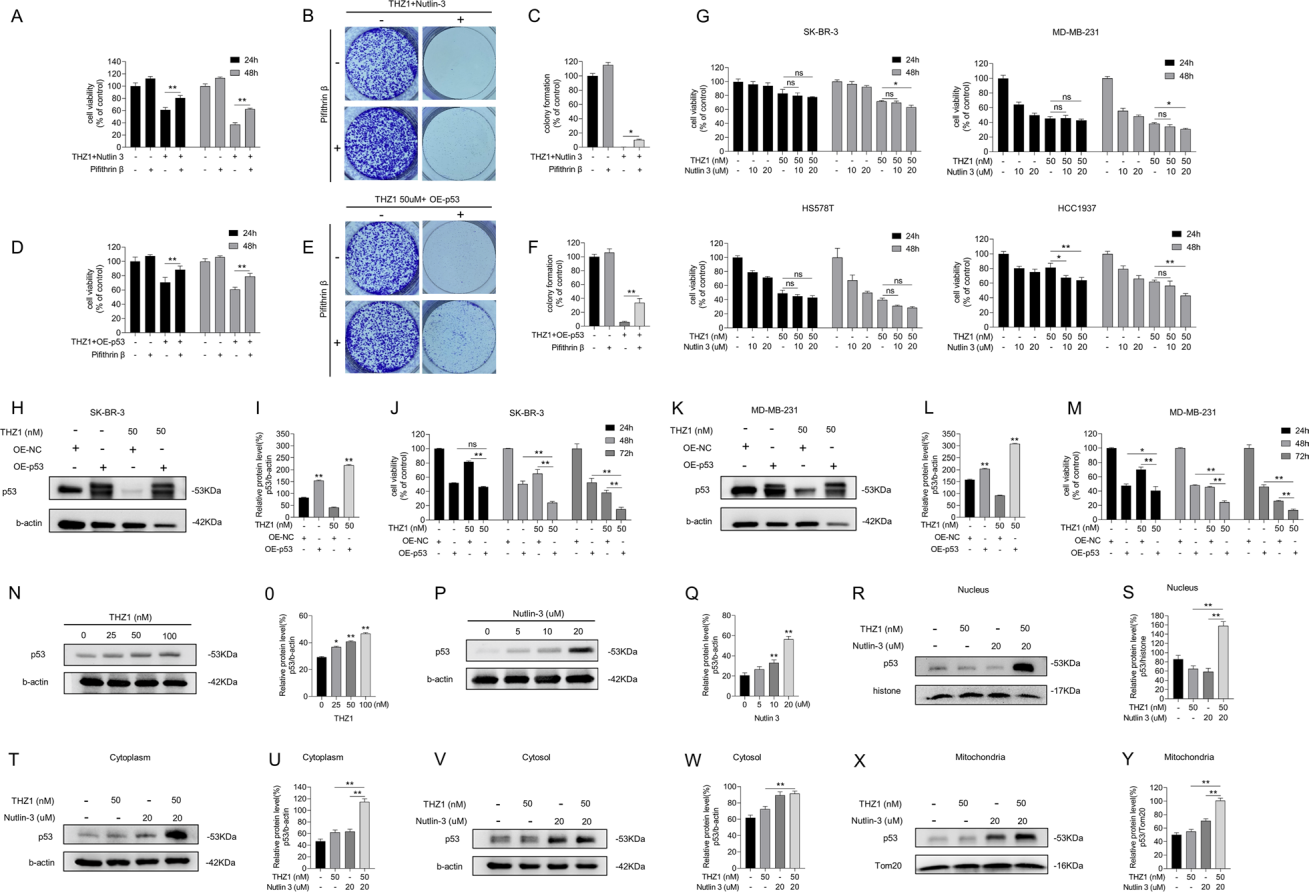
Lethality mediated by nutlin-3 + THZ1 required for effective p53. **A** MCF-7 cell was firstly given PFT β for 6 h, then treated with 50 M THZ1and 20uM nutlin-3. CCK8 assay was used to measure the 24 h or 48 h cell viability after combination was added. **B** Colony formation ability measurement of MCF-7 cell after a 6 h PFT β pretreatment, then added with 50 nM THZ1 + 20uM nutlin-3. **C** Quantitation of colony formation ability in B. **D** MCF-7 cell was firstly transfected with WT p53 plasmids or plasmids of control. After a 6 h incubation with PFT β, 50 M THZ1 was added into the medium of MCF-7 cell with WT p53 plasmids. CCK8 assay was used to measure the 24 h or 48 h cell viability after THZ1 was added. **E **Colony formation ability measurement of MCF-7 cell with WT p53 plasmids or plasmids of control, then treated with 50 nM THZ1. **F** Quantitation of colony formation ability in E. **G** CCK8 assay was used to measure the cell viability of SK-BR-3, MDA-MB-231, HS578T, HCC1937 cells under 50 M THZ1and 20uM nutlin-3 treatment for 24 h or 48 h respectively. **H** Western blot was meant to detect p53 and b-actin protein levels in SK-BR-3 cell which was treated with 50 nM THZ1 for 48 h after WT p53 plasmids transfection. **I** Quantity of P53 in H. **J** CCK8 assay was used to measure the cell viability of SK-BR-3 cell under 50 M THZ1 treatment for 24 h or 48 h respectively. **K** Western blot was meant to detect p53 and b-actin protein levels in MDA-MB-231 cell which was treated with 50 nM THZ1 for 48 h after WT p53 plasmids transfection. **L** Quantity of P53 in K. **M** CCK8 assay was used to measure the cell viability of MDA-MB-231 cell under 50 M THZ1 treatment for 24 h or 48 h respectively. **N**, **O**, **P**, **Q** Western blot was conducted to detect p53 protein levels in MCF-7 cell which was treated with increasing concentrations of THZ1 or nutlin-3 for 48 h. **R**, **S**, **T**, **U**, **V**, **W**, **X**, **Y** Western blot was conducted to detect p53 protein levels in nucleus, cytoplasm, cytosol or mitochondria of MCF-7 cell which was treated with 50 nM THZ1or 20uM nutlin-3 or combination for 48 h. Statistically significant (*P* < 0.05) are *, (*P* < 0.01) are **

It remains unclear whether p53 protein expression or activity is required for the lethal effects of nutlin-3 and THZ1. We utilized a functional p53 interfering agent, PFT β, which reversibly blocks p53-dependent transcriptional activation without affecting p53 mRNA levels. We observed a consistently weaker survival suppression in nutlin-3 and THZ1-treated breast cancer cells when PFT β was added, compared with cells treated with nutlin-3 and THZ1 alone (Fig. 5A–C). The same procedure was repeated for the MCF-7 cells exogenously treated with p53. As shown in Fig. 5D–F, although p53 expression was increased at the gene level, the tumor-suppressive effects were attenuated due to the inactivation of the p53 pathway. Thus, we propose that active p53 is vital in nutlin-3 and THZ1-mediated breast cancer cell survival. When p53 transcription or activity is impaired, it protects breast cancer cells from the lethal effects of the combined treatment.

The viability of different breast cancer cells cotreated with MT p53, nutlin-3, and THZ1 was determined. The tumor-suppressive effects of the cotreatment were not superior to the superimposed effect (Fig. 5G). We determined the viability of MDA-MB-231 cells treated with different concentrations and proportions of nutlin-3 and THZ1 and calculated the CI. The CI was approximately 1, suggesting that this combination exerted an additive effect on MDA-MB-231 cells (Additional file 2: Fig. S2A and B). The effect of nutlin-3 and THZ1, in combination or alone, on the MDA-MB-231 cell cycle was also examined (Additional file 2: Fig. S2C). The cell cycle was arrested in the G2 phase in the combined treatment cell group, with a negligible difference between either treatment group. Thus, either nutlin-3 or THZ1 can induce MDA-MB-231 cell cycle arrest at the G2 phase, and their combination is not required to enhance cell cycle arrest.

Plasmids expressing WT p53 were introduced into SK-BR-3 and MDA-MB-231 cells to restore p53 expression. Immunoblot results confirmed functional p53 expression in these cells (Fig. 5H, I, K, and L). Cell viability analysis revealed that MT p53-carrying breast cancer cells with functional p53 were vulnerable to THZ1 (Fig. 5J, M). Hence, the prominent lethal effect of nutlin-3 and THZ1 in breast cancer-derived cells may depend on functional p53 and an intact p53 pathway.

Expression of p53 in MCF-7 cells was induced by treating with increasing concentrations of THZ1 or nutlin-3 (Fig. 5N–Q). It has been reported that p53 induces cell death pathways by localizing to specific organelles. Therefore, we investigated the accumulation of p53 in cells. Nuclear and cytoplasmic proteins were extracted and blotted separately. When cells were treated with nutlin-3 and THZ1 (Fig. 5R–U), p53 protein levels in the nucleus and cytoplasm significantly increased. As p53 is a crucial factor in initiating mitochondria-associated programmed cell death, we isolated the mitochondria from the cytoplasm to quantify p53 in the cytosol versus mitochondria, using Tom20 as a reference. Interestingly, compared with the cytosol, where p53 protein levels were similar among groups, p53 expression in the mitochondria was enhanced under combined nutlin-3 and THZ1 treatment (Fig. 5V–Y). Therefore, the lethal effects induced by nutlin-3 and THZ1 may be responsible for p53 accumulation in the nucleus and mitochondria.

### Disruption of the transcriptional process, but not CDK7 inhibition, was required for nutlin-3 + THZ1 mediated apoptosis

**Fig. 6:**
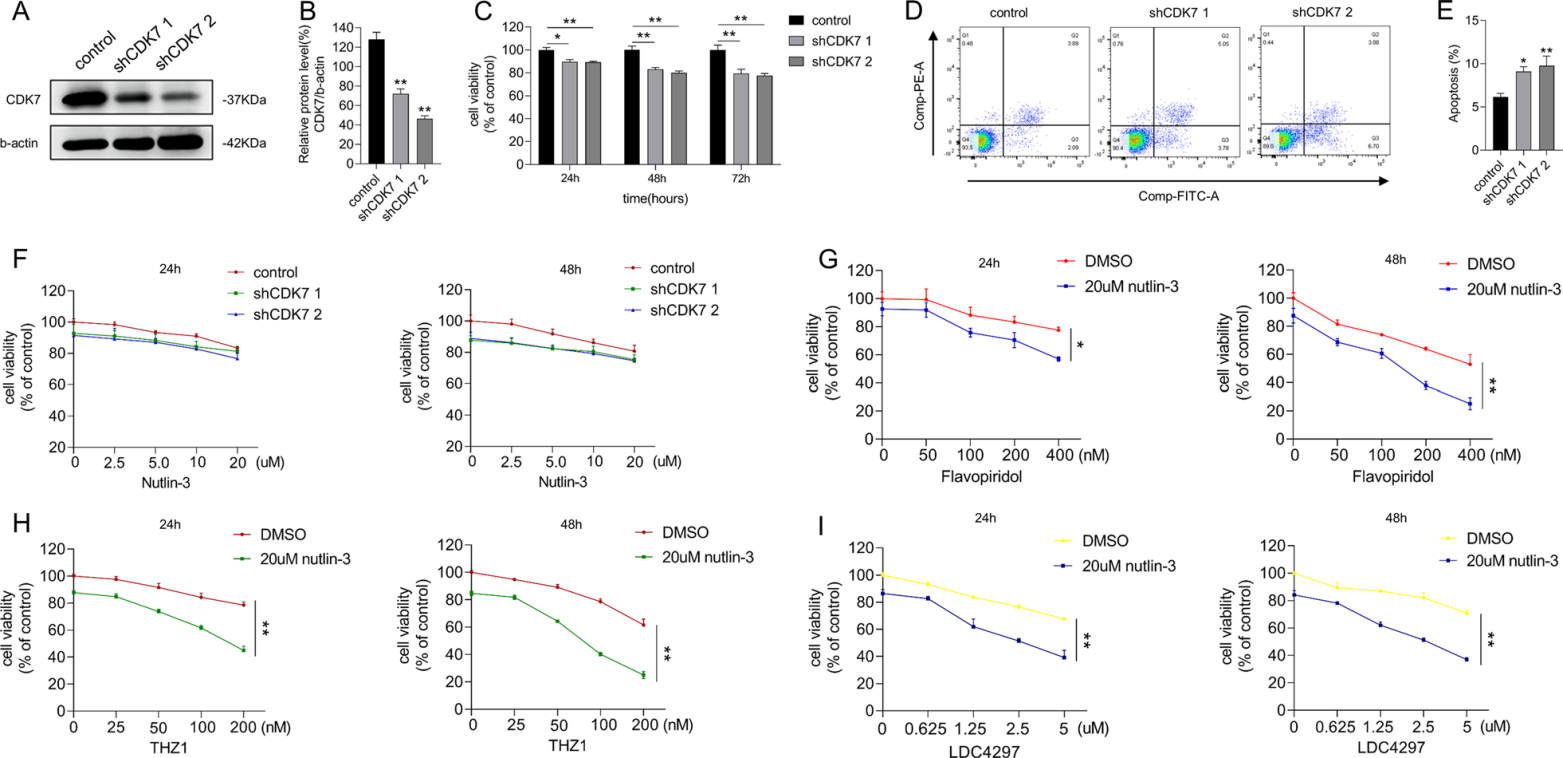
Disruption of transcriptional process, but not CDK7 inhibition, was required for nutlin-3 + THZ1 mediated apoptosis. **A** Western blot was utilized to verify the silencing effects of CDK7 shRNA plasmids in MCF-7 cell. **B** Quantity of CDK7 in A. **C** CCK8 assay was utilized to measure the viability of MCF-7 cell with CDK7 shRNA at 24 h, 48 h and 72 h. **D** Apoptosis was analyzed by flow cytometry after MCF-7 cell was treated with CDK7 shRNA and control shRNA. **E** Quantity of apoptosis in D. **F** CCK8 assay was used to detect the cell viability of MCF-7 cell with control shRNA or CDK7 shRNA under increasing concentrations of nutlin-3 treatment for 24 h or 48 h separately. **G** CCK8 assay was used to detect the cell viability of MCF-7 cell under increasing concentrations of flavopiridol treatment combining 20uM nutlin0-3 for 24 h or 48 h separately. **H** CCK8 assay was used to detect the cell viability of MCF-7 cell under increasing concentrations of THZ1 treatment combining 20uM nutlin0-3 for 24 h or 48 h separately. **I** CCK8 assay was used to detect the cell viability of MCF-7 cell under increasing concentrations of LDC4297 treatment combining 20uM nutlin0-3 for 24 h or 48 h separately. Statistically significant (*P* < 0.05) are *, (*P* < 0.01) are **

As THZ1 is a highly selective covalent CDK7 inhibitor, we replaced THZ1 with lentiviral vectors to deliver shRNA and inhibit CDK7 expression in MCF-7 cells (Fig. 6A, B). Targeted inhibition of CDK7 affected cell proliferation but did not trigger apoptosis (Fig. 6C–E). Next, we treated the cells, with shRNA-decreased CDK7 expression, with 20 µM of nutlin-3; surprisingly, CDK7 inhibition combined with nutlin-3 did not exhibit significant anticancer effects compared with combined nutlin-3 and THZ1 treatment (Fig. 6F). We assumed that this might depend on the treatment sequence and verified this by adding nutlin-3 and THZ1 sequentially. One cell group was pretreated with nutlin-3 for 6 h, and their culture medium was replaced with the fresh medium containing nutlin-3 and THZ1. Another group was pretreated with THZ1 for 6 h before substituting the culture medium with the fresh containing nutlin-3 and THZ1. Although we observed some cell inhibition after 24 h incubation, the difference between the two groups was minimal (data not shown).

THZ1 obstructs gene transcriptional processes by repressing the mitotic cell cycle, and combined nutlin-3 and THZ1 treatment interfere with HCT116 cells by disrupting transcription [7, 23] We hypothesized that nutlin-3 and THZ1-mediated lethality of breast cancer cells depends on extensive transcriptional interference. We verified this hypothesis by assessing the viability of MCF-7 cells treated with flavopiridol, a broad-spectrum competitive CDK inhibitor, and nutlin-3. We found that a dose of flavopiridol exceeding 100 nM induced a gradual synergistic lethal effect. (Fig. 6G). We further modified the dose of THZ1, combined it with 20 µM nutlin-3 to treat MCF-7 cells, and quantified the combined tumor-inhibiting efficiency. MCF-7 cells were more vulnerable on 100 nM THZ1 than 50 nM or lower (Fig. 6H). When the cells were treated with another small-molecule inhibitor with CDK7 affinity, LDC4297, its increasing concentrations caused a gradient reduction in cell viability (Fig. 6I). These data imply that suppression of breast cancer cells with nutlin-3 and THZ1 is necessary to impede transcription.

We treated MCF-7 cells with the polymerase II inhibitor triptolide in the presence or absence of nutlin-3 and examined cell viability (Additional file 3: Fig. S3B and E). We found that adding triptolide facilitated the nutlin-3-induced tumor-suppressive effect. Conversely, there was little difference in MCF-7 cell suppression between the cell groups treated with polymerase I and III inhibitors, CX5461, and ML60218 with or without nutlin-3 (Additional file 3: Fig. S3A, C, D, E, respectively). These results agree with Kalan et al. that blocking transcription is necessary for the synthetic lethality of nutlin-3 and THZ1 [23]. We further verified this mechanism in WT p53 breast cancer cells.

### Nutlin-3 + THZ1 potentiated GSDME cleavage

**Fig. 7:**
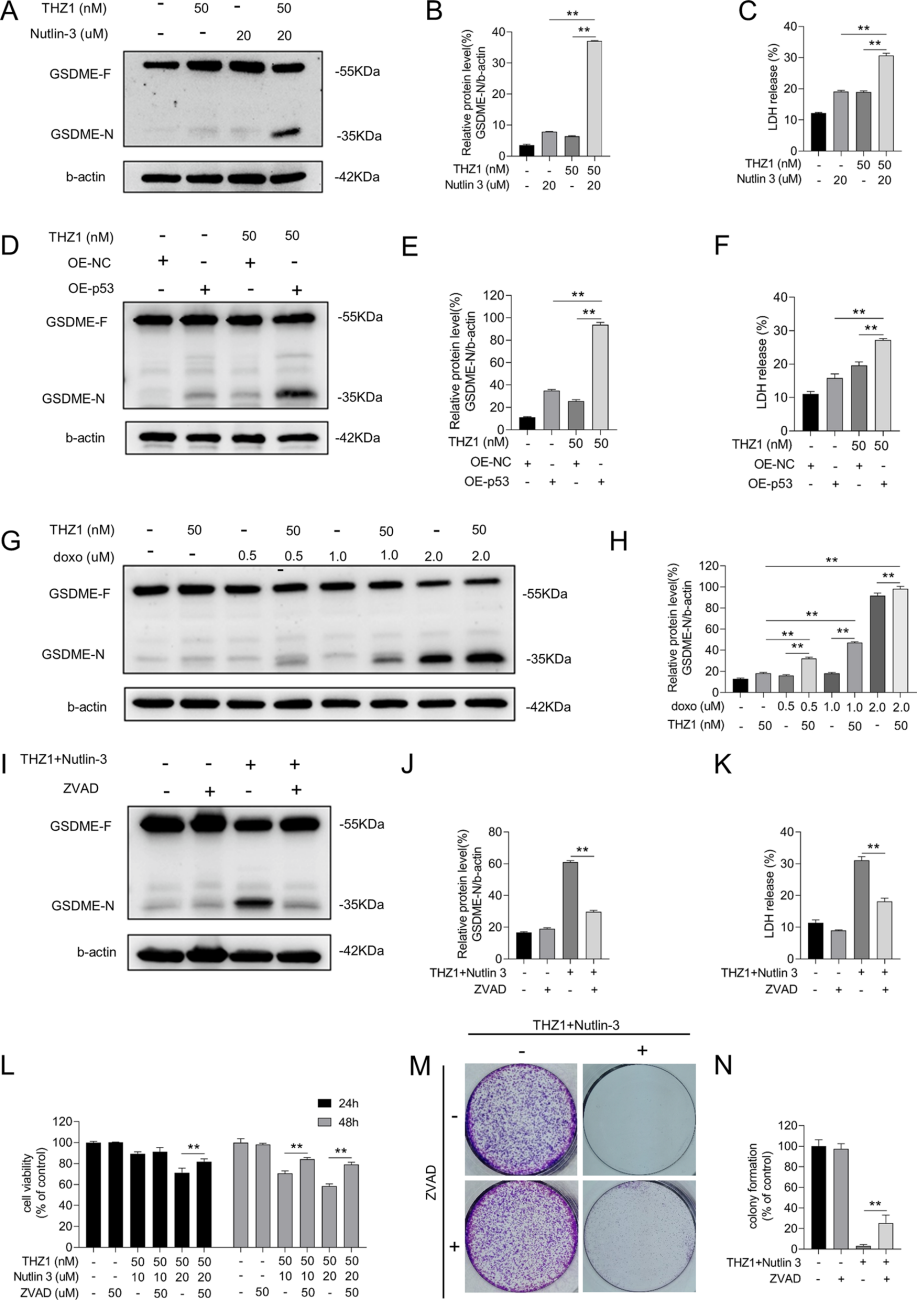
Nutlin-3 + THZ1 potentiated GSDME cleavage. **A** Western blot was meant to detect GSDME-N protein levels in MCF-7 cell which was treated with 50 M THZ1and 20uM nutlin-3 for 48 h. **B** Quantity of GSDME-N in A. **C** The release of LDH was measured after MCF-7 cells treated with 50 nM THZ1 + 20uM nutlin-3 for 48 h. **D** Western blot was meant to detect GSDME-N protein levels in MCF-7 cell which was treated with 50 nM THZ1 for 48 h after WT p53 plasmids transfection. **E** Quantity of GSDME-N in D. **F** The release of LDH was measured after MCF-7 cells treated with 50 nM THZ1 for 48 h after WT p53 plasmids transfection. **G** MCF-7 cell was treated with 50 nM THZ1 combining increasing concentrations of doxorubicin for 48H. The protein level of GSDME-N was detected by western blot. **H** Quantity of GSDME-N in G. **I** MCF-7 cell was co-incubated with ZVAD for 6 h, then treated with 50 nM THZ1 + 20uM nutlin-3 for 48H. The protein level of GSDME-N was detected by western blot. **J** Quantity of GSDME-N in I. **K** The release of LDH of MCF-7 cell which firstly incubated with ZVAD for 6 h, then treated with 50 nM THZ1 + 20uM nutlin-3 for 48H. **L** MCF-7 cell was firstly given ZVAD for 6 h, then treated with 50 M THZ1and 20uM nutlin-3. CCK8 assay was used to measure the 24 h or 48 h cell viability after combination was added. **M** Colony formation ability measurement of MCF-7 cell after a 6 h ZVAD pretreatment, then added with 50 nM THZ1 + 20uM nutlin-3. **N** Quantitation of colony formation ability in M. Statistically significant (*P* < 0.05) are *, (*P* < 0.01) are **

GSDME, a member of the GSDM family, modulates secondary apoptosis by releasing its N-terminal domain that binds to the cytomembrane. The assembly of GSDME N-terminal domains enhances membrane permeability, leading to cell swelling and osmotic lysis. We verified whether GSDME induced cell death using a combination of nutlin-3 and THZ1. Elevated GSDME cleavage was detected in cells cotreated with THZ1 and nutlin-3, WT p53 plasmid transfection, or doxorubicin (Fig. 7A, B, D, E, G, and H). In addition, the amount of released LDH increased (Fig. 7C, F). As caspase-3 activation initiates GSDME cleavage [26, 27], we pre-added a pan-caspase inhibitor, ZVAD, to repress caspase cascade activation induced by the combined nutlin-3 and THZ1 treatment. The inhibitor blocked GSDME cleavage and reduced LDH leakage (Fig. 7I–K). Moreover, ZVAD protected MCF-7 cells from the anticancer effect of nutlin-3 and THZ1, with enhanced proliferation and greater clonal spots (Fig. 7L–N).

## Discussion

The high incidence of breast cancer poses an undeniable threat to millions of women worldwide. Although multiple pathways have been devised to reduce breast cancer development, many unanswered questions remain. Currently, research on small-molecule inhibitors for cancer treatment is gaining attention. For example, THZ1, a covalent CDK7 inhibitor, has a suppressive effect on many cancer cells. Additionally, it was reported to downregulate oncogene-associated super-enhancer-driven transcription, including SOX9 and MYC, which might induce disruption of TNBC transcriptional addiction [28, 29]. We cultured different breast cancer cell lines and treated them with THZ1. The results showed that the inhibitor exerted measurable anti-tumorigenic effects in multiple breast cancer cell lines (Fig. 1). Furthermore, cells expressing MT p53 showed greater sensitivity to THZ1 than those expressing WT p53. We attribute this observation to the different p53 phosphorylation levels induced by CAK. As earlier mentioned, p53, which carries a mutation at a specific site, acts as a potent and efficient phosphorylation substrate [30]. Most mutant p53 lose its original function; however, some p53 mutations have a dominant-negative impact on WT p53 activity or acquire new oncogenic functions [31]. We previously showed that upon CDK7 inhibition, p53 protein levels depend on the protein status. In this study, the wild-type protein expression increased following CDK7 inhibition, whereas that of the mutated decreased [14, 15]. We also showed that CDK7 inhibition induced p53 protein expression in WT p53 breast cancer cells and decreased p53 protein levels in MT p53 breast cancer cells (Figs. 2 and 5). Decreasing mutant p53 protein expression by phosphorylation to abrogate cancer development is a possible strategy to fight cancer [14, 32]. THZ1 had a considerable suppressive effect on MT p53 in breast cancer cells, possibly due to the THZ1-promoted attenuation of MT p53 protein. Conversely, the survival of WT p53 breast cancer cells was affected less. We hypothesized that a significant anticancer effect requires the wild-type p53 protein and its specific threshold level. Indeed, a relatively low amount of WT p53 does not cause meaningful cancer recession (Figs. 1E, F and 5N, P; Additional file 1: S1A) [14, 15].

As this study included different breast cancer cell lines, other genomic alterations, in addition to p53, should be discussed. We mined the ATCC database for mutations and found some of the common mutations in cancer cells were listed. We also searched existing publications but found few studies demonstrating an association between certain common genomic alterations in breast cancer and CDK7. Phosphatidylinositol-4,5-biphosphate 3-kinase catalytic subunit alpha (PIK3CA) participates in the PI3K/AKT/m TOR pathway by mediating cell proliferation, survival, migration, and vesicular trafficking. The MCF-7 cell line was identified to have a PIK3CA mutation (Table 1). CDK7 is not directly connected to the PIK inhibitor, but its downstream factor CDK4/6 has been used in clinical trials with PIK inhibitor alpelisib. According to SOLAR-1 (NCT02437318) and BYLieve (NCT03056755, cohort A) trials, breast cancer patients carrying PIK3CA mutations who received any CDK4/6 inhibitor and fulvestrant treatment followed by alpelisib had a better prognosis [33]. Rat sarcoma virus (Ras), a member of the small GTPase superfamily, regulates cell growth, differentiation, and survival by recruiting and activating downstream effectors, including factors involved in AKT and ERK pathways. The MDA-MB-231 cell line has been reported to have a Ras mutation (Table 1). As previously described, constructive dominant-negative Ras inhibited phenylephrine-induced CDK7 elevation, suggesting that Ras-activated expression requires the regulation of CDK7 or phosphorylation of the C-terminal domain of RNA polymerase II [34]. Furthermore, the physical interaction between Ras GTPase-activating protein (RasGAP) and filamin C facilitates the interaction between RasGAP SH3 domain-binding protein and CDK7 mRNA, which helps increase CDK7 protein expression and mRNA stabilization [35]. However, except for p53, the impact of genomic aberrations in breast cancer cell lines on sensitivity to THZ1 or other CKD7 inhibitors is still unclear. Thus, a deeper exploration of interactions between CDK7 and the above mutations is necessary.

Due to the numerous roles of CDK7 in cell cycle control and transcription, the THZ1 anticancer mechanism proposed in this study can be summarized in two ways. First, THZ1 causes cell cycle arrest by blocking the CAK function of CDK7 to activate other CDK members essential in cell mitosis. Second, THZ1 downregulates oncogene transcription in the super-enhancer area by inhibiting CDK7 [6, 36]. Kalan et al. demonstrated that as long as the p53 transcriptional program was activated by 5-fluorouracil or nutlin-3, the covalent CDK7 inhibitor THZ1 reverted survival repression to cell death [23]. We added THZ1 to MCF-7 cells synchronously given nutlin-3 or doxorubicin or previously introduced to exogenous WT p53 and detected cell viability, colony formation ability, apoptotic cell rate, and protein variation (Figs. 1 and 3). Moreover, the combined effect of nutlin-3 and THZ1 repeated in multiple breast cancer cell lines (Figs. 2 and 5G). Consistent with the results of Kalan et al., when effective p53 was stabilized, breast cancer cells expressing WT p53, not MT p53, were more vulnerable to THZ1. We further explored this observation by transfecting MT p53 breast cancer cells with WT p53 plasmids and determined the degree of PARP cleavage and cell viability under THZ1 treatment. Interestingly, the THZ1 tumor-suppressive effects were amplified (Fig. 4). Although in vivo data are lacking, these findings indicate that patients carrying wild-type p53 in primary breast tumors may benefit from the combined drug treatment.

Although p53 is required for cancer cell death on the combined drugs, the site of its accumulation during cell death is unknown. The protein participates in modulating various genes involved in cell cycle arrest, apoptosis, DNA repair, metabolism, autophagy, and ferroptosis in response to various cellular stresses. Many studies have shown it induces transcription-independent apoptotic pathways by accumulating in different organelles [37–39]. While p53 generally regulates proapoptotic factor transcription in the nucleus, it can also directly interact with them in mitochondria. The interaction triggers mitochondrial outer membrane permeabilization (MOMP) and subsequently mitochondrial programmed cell death [39]. We performed an immunoblot assay and found p53 accumulated in the nucleus and mitochondria of WT p53 breast cancer cells (Fig. 5L and O). Hence, the transcription of proapoptotic factors and MOMP may play a role in nutlin-3 and THZ1-mediated cell death. The p53 mutation has little effect on mitochondrial translocation of p53, and its ability to induce apoptotic signals [40, 41]. Therefore, the competence of WT p53 to transport and modify may be intact in MT p53 cancer cells. When WT p53 protein is expressed in tumors with MT p53, its movement to different cellular organelles and appropriate modifications are initiated and may explain why the tumor-suppressive effects were elevated after transfecting MT p53 breast cancer cells with WT p53 plasmids (Fig. 5F–I).

How p53 protein levels increase in the mitochondria and nucleus after nutlin-3 and THZ1 treatment remains an open question. We speculated p53 accumulates in the nucleus and phosphorylates before translocating to the mitochondria. Several selective CDK7 inhibitors downregulate MDM2 mRNA expression and decrease Mdm2 ubiquitin ligase function by inducing MDM2 ribosomal protein L11 complex formation [10, 42, 43]. Additionally, the selective cyclin-dependent kinase inhibitor roscovitine abolishes the activating phosphorylation of CDK2 and CDK7 at Ser164/170, while it activates WT p53 by inducing phosphorylation at Ser46 [44]. Reactive oxygen species amplify the p53 phosphorylation at Ser46 to facilitate the protein interaction with prolyl-isomerase Pin1. Interestingly, Pin1, identified as an intermediator in p53 mitochondrial translocation, regulates p53 function by specifically binding to p53 phosphorylation sites [45]. Moreover, transcriptional blockade induces p53 translocation to mitochondria, triggering p53-dependent apoptosis [46]. Thus, THZ1 increases nuclear and mitochondrial p53, possibly owing to its transcriptional obstruction effect and p53 phosphorylation at Ser46.

Doxorubicin, a first-line chemotherapeutic drug administered to breast cancer patients, elevates p53 expression by causing DNA damage [47] (Fig. 3H). This drug was hence chosen as the THZ1 partner to treat MCF-7 cells. Indeed, they showed decreased survival when treated with doxorubicin and THZ1 than with either drug alone (Fig. 3I-P). Furthermore, the effect of doxorubicin and THZ1 or doxorubicin and LDC4297 on breast tumor survival was also p53-dependent (Fig. 4H-N). These data suggest that THZ1 and LDC4297 enhance the anticancer effects of chemotherapeutic drugs.

We further explored the role of CDK7 in suppressing nutlin-3 and THZ1-induced breast cancer cell survival. MCF-7 cells with downregulated CDK7 barely responded to the nutlin-3 lethality (Fig. 6H). This finding implies that CDK7 inhibition is dispensable for cancer repression mediated by nutlin-3 and THZ1. We hypothesized that the nutlin-3 and THZ1 lethality depends on intact transcription. As shown in Fig. 6I, an agent with multiple modes of CDK inhibition restrained cell viability more when combined with nutlin-3. These results strongly suggest that extensive transcriptional process disruption, rather than CDK7 inhibition, is necessary for nutlin-3 and THZ1-induced breast cancer cell destruction. Because THZ1 and LDC4297 displayed an affinity for other CDKs at high concentrations, MCF-7 cells showed greater fragility with the addition of a high dose of CDK7 inhibitors compared with a low, which might also imply that broad transcription interference is required for nutlin-3 and THZ1 combination lethality (Fig. 6J, K).

Limited by the experimental conditions, this study was performed in only a few breast cancer cell lines in vitro. Data on tumor–microenvironmental interactions on nutlin-3 and THZ1 treatment and its adverse effects are lacking. Thus, in vivo or 3D tumor model tests should be conducted to show nutlin-3 and THZ1 breast tumor- suppressive effects comprehensively and rigorously validate the safety of this combination.

Zhou et al. reported that p53, activated by MDM2 inhibitors, induces an antitumor response by upregulating genes associated with IFN-α and IFN-γ responses [48]. They described the tumor-suppressive effects of doxorubicin on p53-dependent induction of IFNλIFN-related genes, which is consistent with our demonstration that effective p53 elevation boosts doxorubicin to repress breast cancer cell survival. Zhou et al. further detected granzyme B overexpression under p53 activation treatment, an important factor in triggering GSDME cleavage [49]. Interestingly, we observed that GSDME cleavage and LDH release increased after stimulation with nutlin-3, the introduction of exogenous p53, or doxorubicin combined with THZ1 (Fig. 7A–E). These synergistic effects were reversed by inducing inhibition of caspase cascade with the ZVAD pretreatment (Fig. 7F–H). Several studies revealed that GSDME modulates secondary apoptosis [26, 27], which occurs after primary apoptosis when dead cells are not eliminated in time. Furthermore, Zhang et al. demonstrated that GSDME cleavage could have a role in anticancer immunity [49]. Elevation of GSDME cleavage in our study suggests that the drug combination is related to cancer immunotherapy. However, the risk of inducing cytokine release syndrome when using this treatment must be considered.

## Conclusion

Increased cell cycle progression is a characteristic of tumors, implying that targeted CDK inhibition may be a meaningful anticancer therapy. However, the insensitivity of WT p53 breast cancer cells to THZ1 causes uncertainty in applying CDK7 inhibitors in breast cancer treatment. We enhanced the anticancer THZ1 effect by combining THZ1 with nutlin-3 or other methods to elevate p53 expression, leading to considerable lethality of breast cancer cells. The suppression of tumor survival by nutlin-3 and THZ1 was p53-dependent and required p53 for broad transcriptional disruption. In addition, GSDME cleavage amplification was detected using a combination of THZ1 and nutlin-3. Collectively, we showed a possible way to improve the antitumor effect of THZ1 and broaden its application to breast cancer cells. After improved efficacy and in vivo safety trials, the drug combination may be the treatment of choice for patients with breast cancer resistant to regular therapies.

## Supplementary Information

**Additional file1**. Figure S1 (related to Figure 1) Nutlin-3 elevated the sensitivity of MCF-7 cells to THZ1. A MCF-7 cells colony formation ability under increasing concentrations of nutlin-3 was measured by colony formation assay. B Cell cycle detection assay was performed on MCF-7 cells treated with THZ1 +/- nutlin-3. C CCK8 assay was utilized to measure MCF-7 cell viability. D CI of nutlin-3+THZ1 on MCF-7 cells. E CCK8 assay was utilized to measure MCF-10A cells viability. F CI of nutlin-3+THZ1 on MCF-10A cells.

**Additional file2**. Figure S2 (related to Figure 5) Lethality mediated by nutlin-3+THZ1 required for effective p53. A CCK8 assay was utilized to measure MDA-MB-231 cell viability. B CI of nutlin-3+THZ1 on MDA-MB-231 cells. C Cell cycle detection assay was performed on MDA-MB-231 cells treated with THZ1 +/- nutlin-3.

**Additional file3**. Figure S3 (related to Figure 6) Disruption of transcriptional process, but not CDK7 inhibition, was required for nutlin-3+THZ1 mediated apoptosis. A CCk8 assay was meant to detect MCF-7 cell viability under Pol I inhibitor CX5461 +/- nutlin-3. B CCk8 assay was meant to detect MCF-7 cell viability under Pol II inhibitor triptolide +/- nutlin-3. C CCk8 assay was meant to detect MCF-7 cell viability under Pol III inhibitor ML60218 +/- nutlin-3. D CCk8 assay was meant to detect MCF-7 cell viability under Pol I inhibitor CX5461 combined with Pol III inhibitor ML60218 +/- nutlin-3. E P53 protein level and cleavage degree of PARP was measured by western blot.

## Data Availability

Not applicable.

## Abbreviations

CDK: Cyclin-dependent kinase
CAK: CDK-activated kinase
RNA Pol II: RNA polymerase II
TNBC: Triple-negative breast cancer
MDM2: Murine double minute-2
WT: Wild type
MT: Mutant type
OE: Overexpressing
IDC: Invasive ductal carcinoma
MOMP: Mitochondrial outer membrane permeabilization
CI: Combination index
LDH: Lactate dehydrogenase
PIK3CA: Phosphatidylinositol-4,5-biphosphate 3-kinase catalytic subunit alpha
RAS: Rat sarcoma
RasGAP: Ras GTPase-activating protein
