# Supplementary figures and images for: Elevation of effective p53 expression sensitizes wild-type p53 breast cancer cells to CDK7 inhibitor THZ1
Source: Cell Commun Signal. 2022 Sep 5;20:96. doi: 10.1186/s12964-022-00837-z (PMC9442925; doi:10.1186/s12964-022-00837-z)

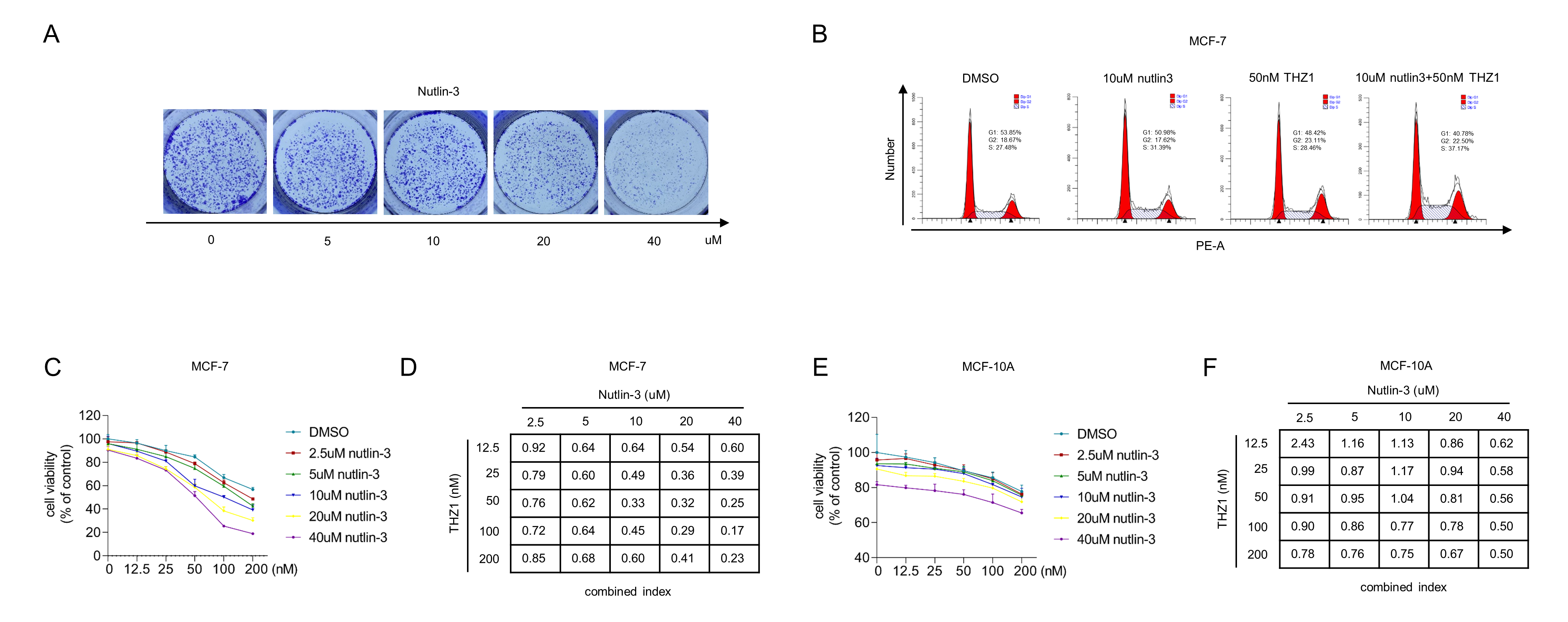

Supplement: Supplementary file 2 — Additional file1. Figure S1 (related to Figure 1) Nutlin-3 elevated the sensitivity of MCF-7 cells to THZ1. A MCF-7 cells colony formation ability under increasing concentrations of nutlin-3 was measured by colony formation assay. B Cell cycle detection assay was performed on MCF-7 cells treated with THZ1 +/- nutlin-3. C CCK8 assay was utilized to measure MCF-7 cell viability. D CI of nutlin-3+THZ1 on MCF-7 cells. E CCK8 assay was utilized to measure MCF-10A cells viability. F CI of nutlin-3+THZ1 on MCF-10A cells. [file 12964_2022_837_MOESM2_ESM.tif]

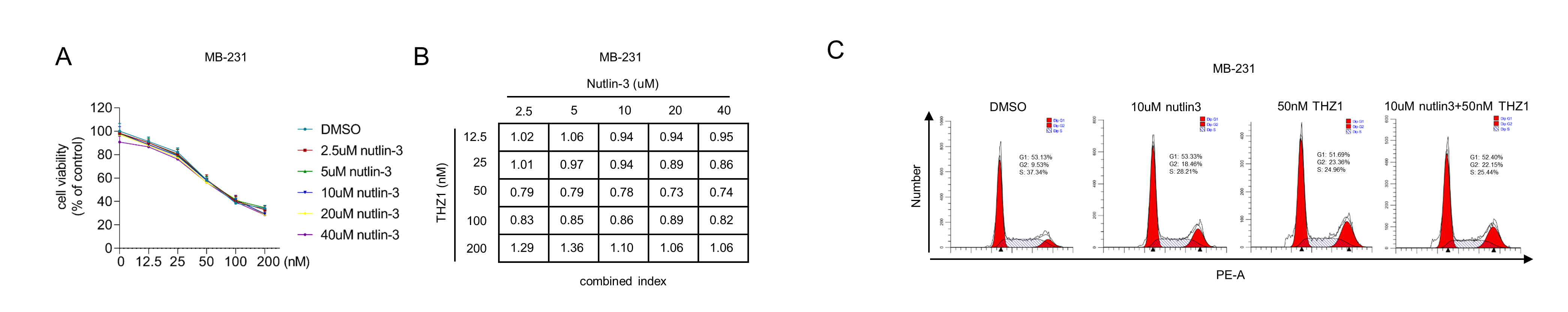

Supplement: Supplementary file 3 — Additional file2. Figure S2 (related to Figure 5) Lethality mediated by nutlin-3+THZ1 required for effective p53. A CCK8 assay was utilized to measure MDA-MB-231 cell viability. B CI of nutlin-3+THZ1 on MDA-MB-231 cells. C Cell cycle detection assay was performed on MDA-MB-231 cells treated with THZ1 +/- nutlin-3. [file 12964_2022_837_MOESM3_ESM.tif]

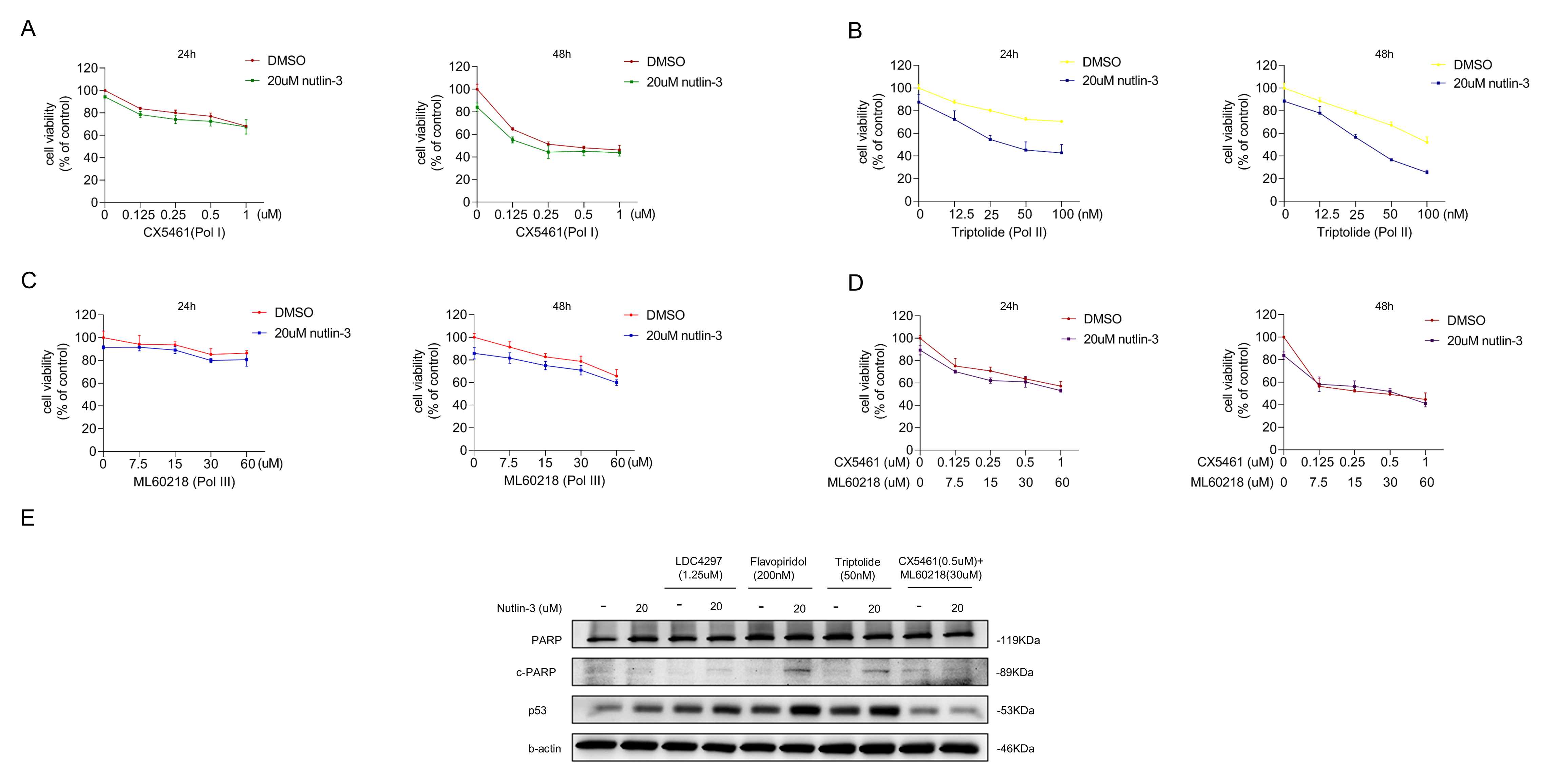

Supplement: Supplementary file 4 — Additional file3. Figure S3 (related to Figure 6) Disruption of transcriptional process, but not CDK7 inhibition, was required for nutlin-3+THZ1 mediated apoptosis. A CCk8 assay was meant to detect MCF-7 cell viability under Pol I inhibitor CX5461 +/- nutlin-3. B CCk8 assay was meant to detect MCF-7 cell viability under Pol II inhibitor triptolide +/- nutlin-3. C CCk8 assay was meant to detect MCF-7 cell viability under Pol III inhibitor ML60218 +/- nutlin-3. D CCk8 assay was meant to detect MCF-7 cell viability under Pol I inhibitor CX5461 combined with Pol III inhibitor ML60218 +/- nutlin-3. E P53 protein level and cleavage degree of PARP was measured by western blot. [file 12964_2022_837_MOESM4_ESM.tif]
